# An in Silico Approach to Reveal the Nanodisc Formulation of Doxorubicin

**DOI:** 10.3389/fbioe.2022.859255

**Published:** 2022-02-25

**Authors:** Daiyun Xu, Xu Chen, Zhidong Chen, Yonghui Lv, Yongxiao Li, Shengbin Li, Wanting Xu, Yuan Mo, Xinpei Wang, Zirui Chen, Tingyi Chen, Tianqi Wang, Zhe Wang, Meiying Wu, Junqing Wang

**Affiliations:** ^1^ School of Pharmaceutical Sciences (Shenzhen), Sun Yat-sen University, Shenzhen, China; ^2^ Department of Pathology, The Eighth Affiliated Hospital, Sun Yat-sen University, Shenzhen, China

**Keywords:** molecular dynamics, Doxorubicin, nanodiscs, prodrugs, Drug delivery, lipidation

## Abstract

Molecular dynamic behaviors of nanodisc (ND) formulations of free doxorubicin (DOX) and DOX conjugated lipid prodrug molecules were investigated by molecular dynamics (MD) simulations. We have unveiled how formulation design affects the drug release profile and conformational stability of ND assemblies. Our simulation results indicate that free DOX molecules loaded in the ND system experienced rapid dissociation due to the unfavorable orientation of DOX attached to the lipid surface. It is found that DOX tends to form aggregates with higher drug quantities. In contrast, lipidated DOX-prodrugs incorporated in ND formulations exhibited sufficient ND conformational stability. The drug loading capacity is dependent on the type of lipid molecules grafted on the DOX-prodrug, and the drug loading quantities in a fixed area of NDs follow the order: DOX-BMPH-MP > DOX-BMPH-TC > DOX-BMPH-PTE. To gain further insight into the dynamic characteristics of ND formulations governed by different kinds of lipidation, we investigated the conformational variation of ND components, intermolecular interactions, the solvent accessible surface area, and individual MSP1 residue flexibility. We found that the global conformational stability of DOX-prodrug-loaded ND assemblies is influenced by the molecular flexibility and lipidated forms of DOX-prodrug. We also found that the spontaneous self-aggregation of DOX-prodrugs with increasing quantities on ND could reduce the membrane fluidity and enhance the conformational stability of ND formulations.

## Introduction

Doxorubicin (DOX), a potent anthracycline cytotoxic drug, has been routinely used as a frontline chemotherapeutic agent in the treatment of various cancers. The anticancer mechanisms of DOX are known to involve multiple mechanisms, including intercalation of DOX into DNA double helix (Coldwell et al., 2008), inhibitions of several molecular targets (topoisomerase enzymes I and II, DNA and RNA polymerase) (Momparler et al., 1976; Foglesong et al., 1992; Marinello et al., 2018), generation of reactive oxygen species (ROS) (Davies and Doroshow, 1986), upregulation of C6 ceramide level (Ji et al., 2010), induction of autophagic cell death (Velez et al., 2011), and immunostimulatory effects (Mattarollo et al., 2011).

However, its clinical utility is restricted by dose-dependent toxicity on noncancerous cells of major organs, including the heart, liver, kidney, and brain (Chatterjee et al., 2010; Octavia et al., 2012; Tacar et al., 2013). Due to these unspecific side effects of conventional DOX therapy, various drug delivery systems (DDSs) such as liposomes, micelles, dendrimers, and inorganic nanoparticles have been developed to enhance therapeutic efficacy minimize the adverse effects of DOX. However, the optimized biocompatibility and pharmacokinetics of these DDSs are critically dependent on PEGylation technology. This approach potentially results in several negative aspects, including restriction of cellular uptake, anti-PEG immune response, non-degradability, and heterogeneity in production. These unfavorable impacts have plagued the clinical translation of most DDS, and hence this has motivated the discovery of alternative PEG-free DDSs.

Endogenous and synthetic lipoproteins, such as high-density lipoprotein (HDL)-like nanodiscs, have rapidly evolved as a promising DDS for small molecules, peptides, and nucleic acids because of their intrinsic long *in vivo* half-lives and passive targeting properties (Kuai et al., 2017; Kuai et al., 2018; Kadiyala et al., 2019). Therefore, nanodisc drug formulation has attracted significant attention in the field of nanomedicine (Lacko et al., 2015; Wang et al., 2018; Tang et al., 2021). Several studies regarding nanodisc-based DOX delivery that employed different encapsulation strategies have shown increased efficacy and reduced side effects in anticancer therapy (Murakami et al., 2010; Yuan et al., 2013; Wang et al., 2014; Kuai et al., 2018). However, nanodisc (ND) formulation of DOX approached by different encapsulation designs can lead to variable drug-loading capacity (DLC), distinct drug release profiles, altered structures, and surface charges, as well as stability of the ND system. These characteristics are critical for DOX-DDS development. Although conventional trial and error approaches may provide sufficient guidance on formulation development, this process is usually sophisticated, time-consuming, and costly (Boruah et al., 2019). Besides, the rational design of DDS formulation depends on a detailed understanding of molecular principles; the mechanistic insights at the molecular level are hard to obtain through experimental research (Ramezanpour et al., 2016). Theoretical studies based on computational modeling methods enable the initial screening of formulation variables to predict potential conditions to accelerate the experimental process (Mehta et al., 2019). In this regard, molecular dynamics (MD) simulations offer a powerful tool to inspect structural, molecular interactions, and dynamic features of drugs and carriers (Bunker and Rog, 2020). Taking into account these considerations, it appears desirable to initially predict the optimum ND formulation of DOX through computational methods.

**FIGURE 1:**
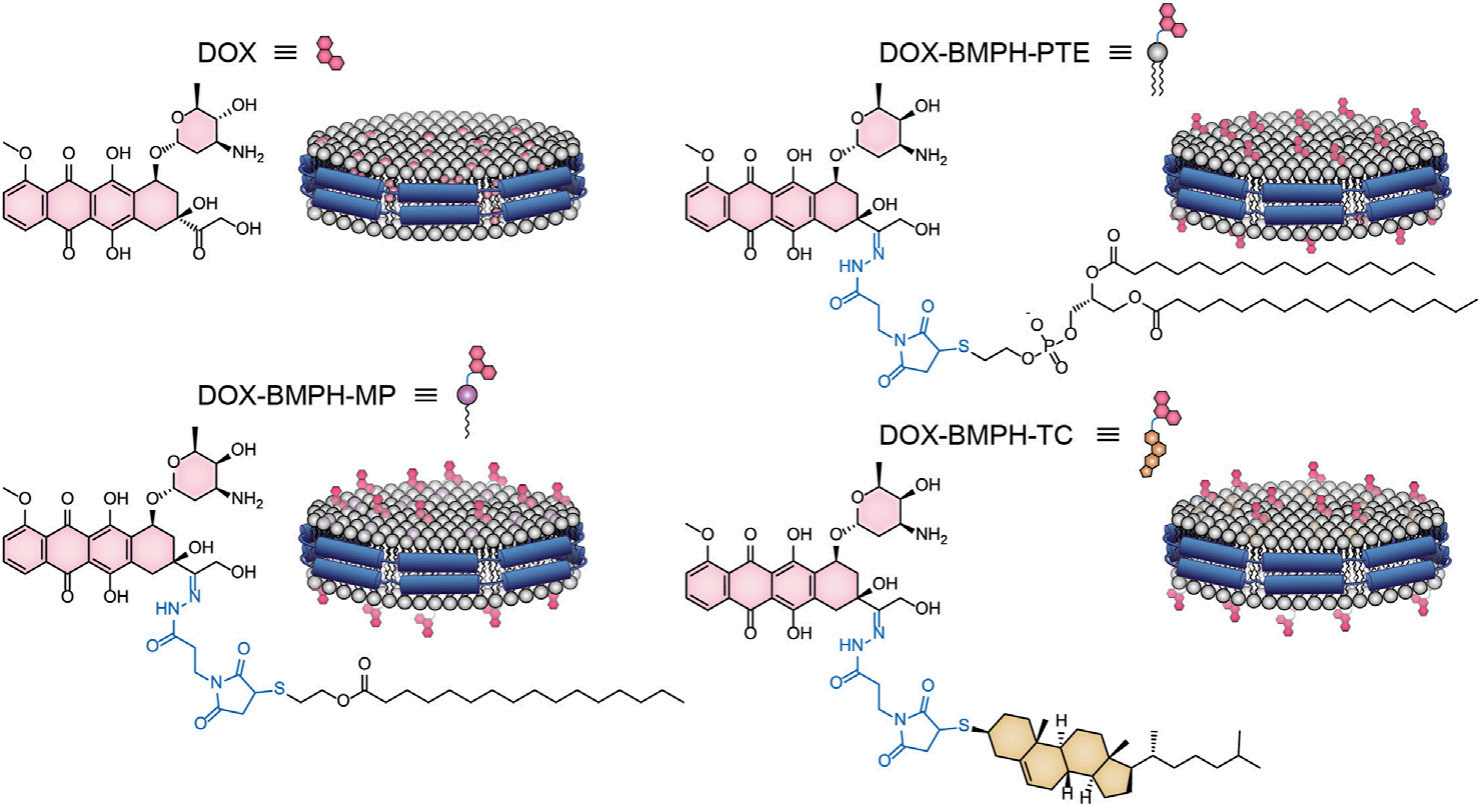
A flow chart summarizes the individual steps for the MD simulation of ND systems.

Here we present an in silico approach that adopted the ND modeling and MD simulation techniques to investigate the effect of different formulation designs on DOX loaded ND DDS (Figure 1). Free DOX and lipidated-DOX prodrugs are designed to access the formulation variables on the structural and dynamical behavior of ND assemblies. The present study focuses on the influence of different lipidated forms of DOX and the drug-to-lipid ratio (D/L ratio) on drug release profile, the structural and dynamic properties of ND-DOX formulations. Moreover, the mechanism of the interaction between DOX and the ND lipid bilayer is poorly understood. This drove us to investigate the behavior of free DOX and lipidated-DOX prodrug molecules in the DPPC bilayer ND through MD simulations.

## Materials and Methods

### Preparation of the Nanodisc System

**FIGURE 2:**
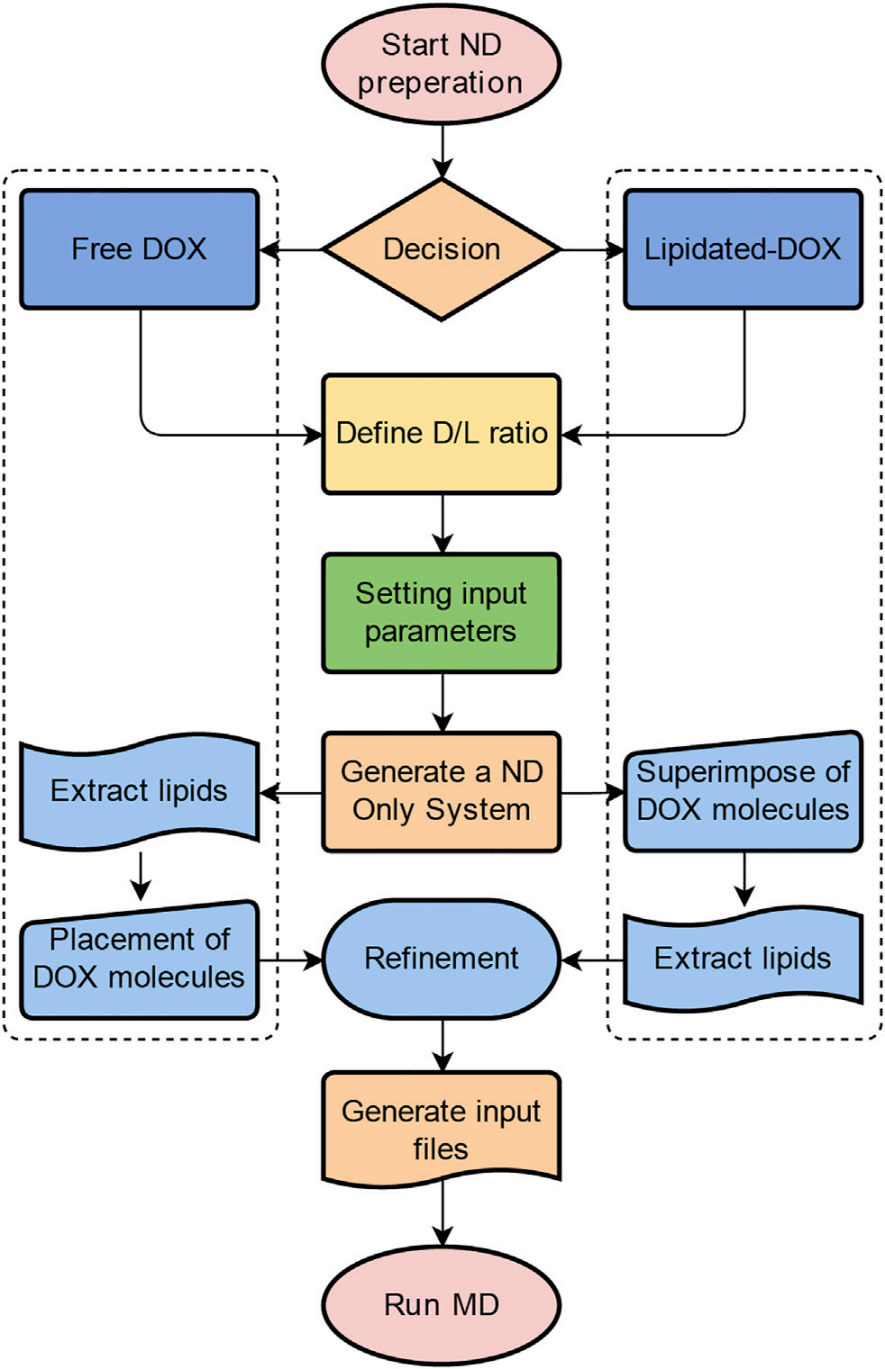
MD simulation results of free-DOX incorporated DPPC ND formulation. The RMSD trajectory of DPPC, MSP1, and ND complex as a function of simulation time in nanoseconds (ns) for **(A)** the empty ND, **(B)** 10% DOX-ND, **(C)** 25% DOX-ND, and **(D)** 50% DOX-ND. **(E)** The RMSD trajectory of DOX molecules with increasing proportions (10, 25, and 50%) as a function of simulation time in ns. The initial and final snapshot of the simulation for each DOX-ND formulation: **(F)** 10% DOX-ND, **(H)** 25% DOX-ND, and **(J)** 50% DOX-ND. The interactions of the DOX molecules with the ND differed depending on the proportions of DOX: **(G)** 10% DOX, **(I)** 25% DOX, and **(K)** 50% DOX.

Membrane scaffold protein 1 (MSP1) and 1,2-dipalmitoyl-sn-glycero-3-phosphocholine (DPPC) were assembled into NDs using CHARMM-GUI (Qi et al., 2019). The diameter of all nanodiscs was 9.8 nm. For preparing four different ND formulation systems: 1. Free DOX, 2. DOX-BMPH-PTE (DOX-N-β-maleimidopropionic acid hydrazide-1,2-Dipalmitoyl-sn-Glycero-3-Phosphothioethanol), 3. DOX-BMPH-MP (DOX-N-β-maleimidopropionic acid hydrazide-2-Mercaptoethyl palmitate), and 4. DOX-BMPH-TC (DOX-N-β-maleimidopropionic acid hydrazide- Thiocholesterol), four corresponding structural analogs (1. Sitosteryl Glucoside, 2. DPPC, 3. Hexadecanoate, and 4. Campesteryl Glucoside) were selected from CHARMM-GUI for subsequent positional labeling. The selected structural analogs were randomly inserted into the ND system with a predefined set of proportions (10, 25, and 50%). Then DOX and Lipidated-DOX were superimposed to the labeled analog using flexible docking, and the labeled analog was subsequently removed. To eliminate the impact of the wrong docking poses on simulation accuracy, only the pose where the DOX sugar moiety is at the hydrophilic interface, and the molecule as a whole is wrapped in the pocket of the membrane layer is preserved. Each step in the process is presented in a flowchart (Figure 2). The control system (empty ND) consists of two MSP1 and 190 DPPCs, and the concrete composition of other ND formulation systems is described in Table 1. Each MSP1 contained 200 residues and a total of 400 residues in each ND system. The system was protonated at 298 K, the salt concentration was set to 0.145 M, and the pH was set to 7.35. All systems were solvated in cubic boxes using TIP3P water and neutralized by adding 0.145 M NaCl. Energy minimization for up to 10,000 steps was performed using the gradient descent algorithm to optimize the system.

**TABLE 1:** The composition of each ND formulation system.

Proportion (number)	DOX (%)		Lipidated-DOX					
	DPPC	DOX	DPPC	DOX*	DPPC	DOX#	DPPC	DOX†
10	180	20	172	18	180	20	180	20
25	156	52	142	48	168	56	156	52
50	116	116	94	96	128	128	116	116

DOX* denotes DOX-BMPH-PTE; DOX# denotes DOX-BMPH-MP; DOX† denotes DOX-BMPH-TC.

### Parameter Settings of Molecular Dynamics Simulations

0.25 ns of NVT equilibration and 1.625 ns of NPT equilibration were performed. System temperature and pressure were maintained at 298 K, and at 1 bar using the Berendsen algorithm, respectively. The Nose-Hoover and Parrinello-Rahman algorithms were used to maintain temperature and pressure in the production simulation (Martyna et al., 1992). The constraints of heavy atoms in the system were gradually released until free in the equilibration stage. The simulation with the time step of 2 fs lasting 100 ns was performed for each system. The CHARMM36 force field was applied to all simulations (Huang and MacKerell Jr, 2013). The LINCS algorithm was used to constrain hydrogen bonds, and Coulomb interactions were calculated using particle-mesh Ewald (Hess et al., 1997). The root-mean-square deviation (RMSD), root-mean-square fluctuation (RMSF), solve accessible surface areas (SASA), and hydrogen bonds of the system were calculated in the trajectory analysis. All structures in the trajectory were clustered based on the RMSD of MSP1, and the structure in the largest cluster with the lowest RMSD relative to all other structures was defined as the central structure. All simulations were performed using GROMACS 2020.5 accelerated by CUDA modules, and all programs ran on AMD Ryzen™ Threadripper™ PRO 3995WX and The GeForce RTX^™^ 3080 Ti graphics card (Van Der Spoel et al., 2005).

## Results and Discussion

### Dynamic Behaviour of DOX Molecules in DPPC ND Formulation

**FIGURE 3:**
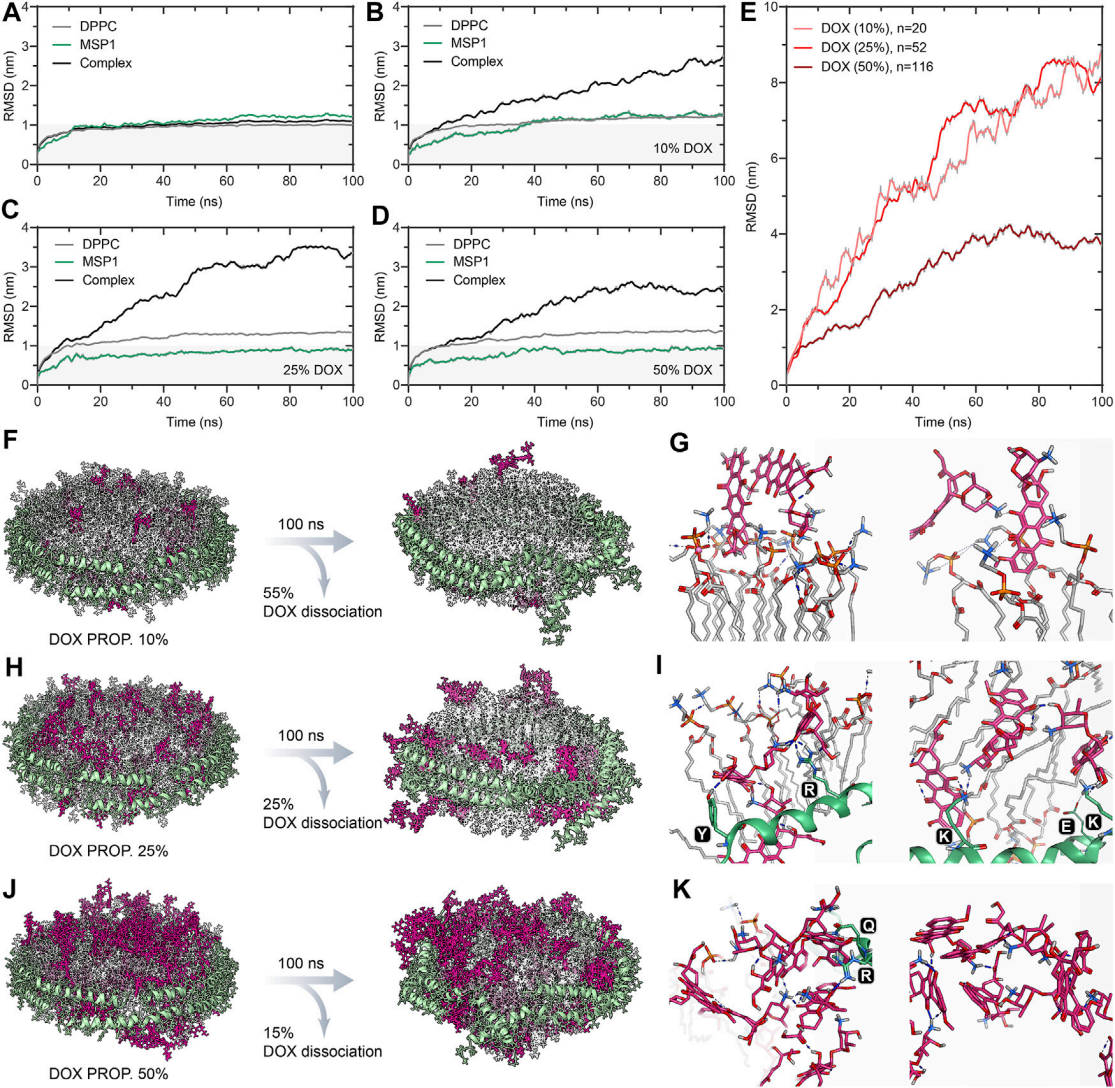
RMSD trajectories and snapshots of center clusters for various types of lipidated-DOX prodrug ND formulations with increasing drug proportions (10, 25, and 50%). **(A)** ND formulation of DOX-BMPH-PTE. **(B)** ND formulation of DOX-BMPH-MP. **(C)** ND formulation of DOX-BMPH-TC.

We initially investigated the MSP1/DPPC ND formulations of free DOX molecules through 100 ns all-atom MD simulations. Three different proportions of DOX ranging from 10 to 25% and 50% of total lipids were randomly placed into the ND bilayer. Considering the structural stability of ND and molecular motion of DOXs in the DPPC membrane, the root-mean-square deviation (RMSD) of DOX, DPPC, MSP (MSP1), and ND complex is determined in Figures 3A–E as a function of time. In the case of an empty ND system (Figure 3A), DPPC and MSP1 reach equilibrium in the first 12 ns of the 100 ns trajectories. Both DPPC and MSP show similar trends with an average RMSD (after 50 ns of simulation) of 1.00 and 1.20 nm, respectively. The global motions of the ND complex are plotted by the RMSD values comprising of DPPC and MSP in the trajectory, with RMSD quickly reaching a plateau of ∼1 nm after about 12 ns of simulation. In contrast to the empty ND system (control), the 10% DOX-incorporated ND assembly was unstable during the 100 ns of simulation (Figure 3B), with RMSD values undergoing a progressive increase of approximately 2.5-fold higher than control at the end of the simulation. However, The average RMSD value (after 50 ns of simulation) of DPPC and MSP1 is 1.18 and 1.22 nm, respectively. This indicates the significant RMSD increment of the ND complex is attributed to the random motion of DOXs.

To further study the role of DOX content in ND stability, ND with elevated DOX content of 25 and 50% were simulated under the same conditions. The structure of the ND complex (25% DOX) was increasingly unstable throughout the simulations, with the highest RMSD above 3.5 nm (Figure 3C). In contrast, the ND complex with higher DOX content (50%) generally shows lower RMSD with a maximum value of ∼2.6 nm (Figure 3D). However, the RMSD values of DPPC display the same increasing trend in 25 and 50% DOX of ND complex by maintaining an average of 1.31 and 1.35 nm during the second half of 50 ns, respectively. This implies the loss of constraints or reduced density between DPPC molecules. Moreover, all simulations show that an increase in the ratio of DOX results in little effect on the RMSD of MSP1. In this regard, the global instability mainly comes from the dissociation of DOX from the ND complexes. Interestingly, the RMSD profiles of 10 and 25% of DOX molecules in ND formulations show a similar trend with increasing RMSD values along the trajectories, while a higher proportion of DOX (50%) in the ND formulation leads to improved global stability, as reflected in lower RMSD pattern of the DOX molecules (Figure 3E). The simulation results suggest the self-aggregation propensity of DOX molecules.

In order to gain further insight into the dynamic behavior of DOX molecules, the first and last snapshots of MD trajectories are illustrated, and DOX-DPPC/MSP1 interactions are analyzed (Figures 3F–K). The drug leakage evaluation shows that 55% of DOX quantities were dissociated from the ND (10% DOX) throughout 100 ns MD simulations, suggesting variable interactions between DOX and DPPC ND bilayer (Figure 3F). However, the degree of DOX dissociation behavior is dropped to 25 and 15%, with increases in DOX-loading content from 25 to 50%, respectively (Figures 3H,J), indicating multiple network interactions were created via homomolecular and heteromolecular interactions. In the case of the low content of DOX (10%), DOX can interact with the DPPC molecules because of its amphiphilic structure. The positively charged amino sugar moiety can readily interact with the negatively charged phosphate group of DPPC through electrostatic interactions. The anthraquinone (AQ) ring of DOX can insert into the DPPC bilayer *via* hydrophobic interaction. In the case of the mid content of DOX (25%) (Figure 3I), additional interactions were formed between the DOX and polar or charged residues in MSP1. Specifically, Arg, Lys, and Glu residues frequently interact with the amino sugar and AQ moieties of DOX molecules. This results in the relocation of DOX molecules toward the ND rim, which is also reflected in the decrease of average RMSD values of MSP1 (Figures 3A–D). In addition to DOX-MSP1 interactions, the high content of DOX (50%) can form a giant network of DOX molecules on the interface of ND *via* both hydrogen bonding and hydrophobic π-π interactions (Figure 3K). This self-aggregation led to an arrested molecular motion and improved association of DOX with the ND. Therefore, we consider that the design of ND formulation involved free DOX molecules should be primarily concerned with the increased self-aggregation tendency at higher DOX concentrations (Fonseca et al., 1997; Fülöp et al., 2013). Such spontaneous homomolecular interactions may distort the orientational-dependent interactions between DOX and ND assembly.

### The Impact of Drug Conjugation on ND Formulation

**FIGURE 4:**
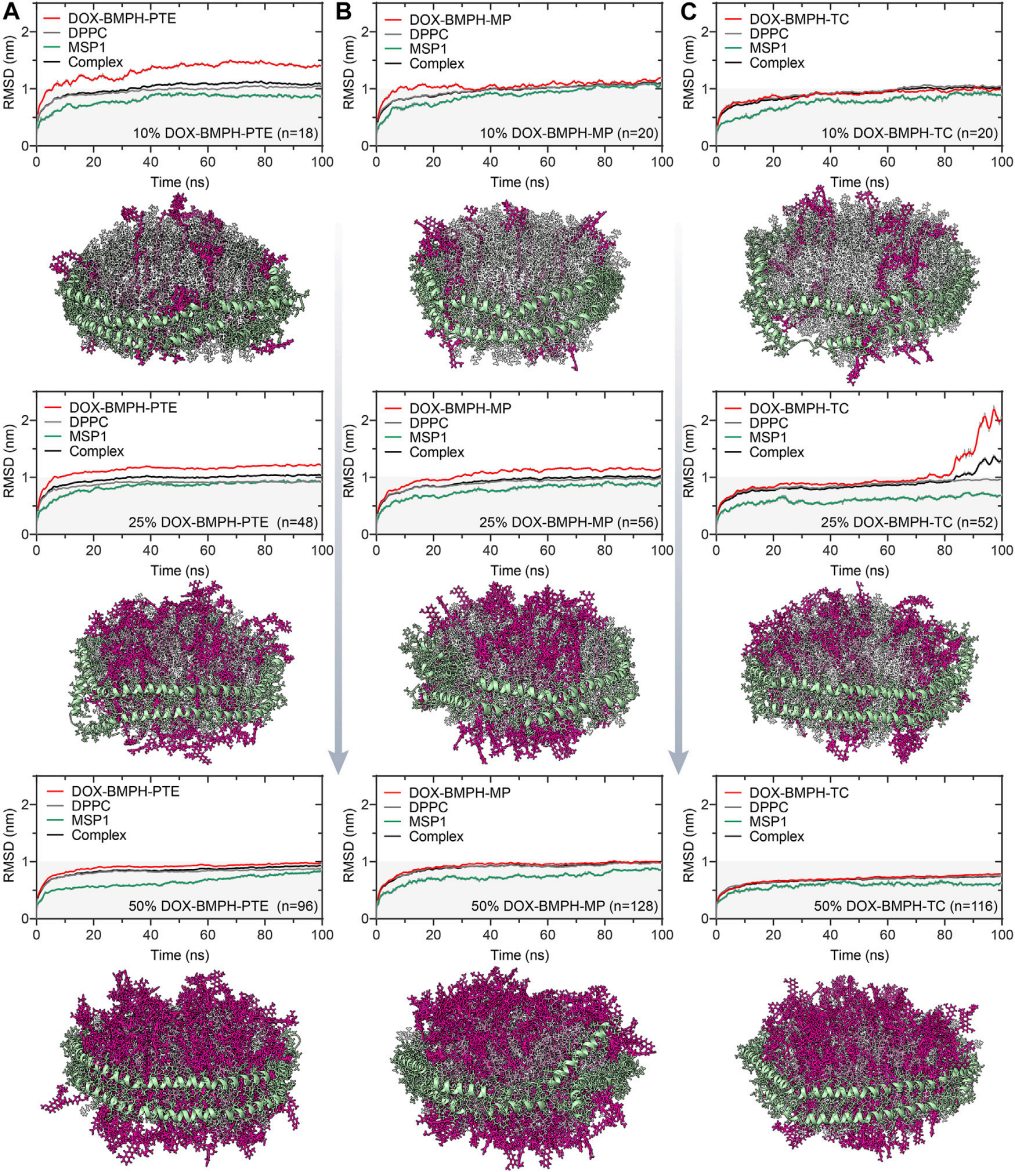
MD simulation analysis of hydrogen bonds formed between DOX prodrug molecules and MSP1 protein in ND complexes. Dynamic measurements of hydrogen bond numbers in ND formulations of **(A)** DOX-BMPH-PTE (10%), DOX-BMPH-MP (10%), and DOX-BMPH-TC (10%). **(B)** DOX-BMPH-PTE (25%), DOX-BMPH-MP (25%), and DOX-BMPH-TC (25%). **(C)** DOX-BMPH-PTE (50%), DOX-BMPH-MP (50%), and DOX-BMPH-TC (50%). Dynamic measurements of hydrogen bonds per drug molecule in ND formulations of **(D) **DOX-BMPH-PTE. **(E)** DOX-BMPH-MP. **(F)** DOX-BMPH-TC. Electrostatic interactions between DOX prodrug molecules and MSP1 protein in ND formulations of **(G)** DOX-BMPH-PTE. **(H)** DOX-BMPH-MP. **(I)** DOX-BMPH-TC.

Three existing types of DOX lipidation are evaluated by a similar MD simulation approach. MD simulation results of ND formulation containing increasing quantities (10, 25, 50%) of BMPH-PTE, BMPH-MP, and BMPH-TC conjugated pH-sensitive DOX prodrugs and their corresponding central structure with the lowest RMSD score in the cluster are illustrated in Figure 4. In the group of ND formulations of DOX-BMPH-PTE (Figure 4A), no DOX leakage was observed during the MD simulations. Visual inspection on the ND complexes showed that DOX-BMPH-PTE molecules were tightly assembled with DPPC NDs. The RMSD profile of each component trajectory remains generally stable after 50 ns of simulations. Lower average RMSD values of DOX-BMPH-PTE and MSP1 are observed with an increase in DOX-BMPH-PTE proportion (Figure 4A). This implies the loss in the degree of ND conformational freedom and plausibly relative to the concentration-dependent DOX self-aggregation and DOX-MSP1 interactions.

Similar dynamic behavior was observed in ND formulations of DOX-BMPH-MP and DOX-BMPH-TC. It is worth noting that each ND assembly possessed the same initial surface area, and the packing capacity is dependent on the surface area per lipid molecule. In this regard, the DOX-BMPH-PTE molecule occupies the largest area (63.0 Å^2^) on the ND among the simulated prodrugs, followed by DOX-BMPH-TC (40 Å^2^) and DOX-BMPH-MP (30 Å^2^). Therefore, ND formulated DOX-BMPH-MP appears to have the highest packing capacity. However, a parallel comparison between DOX-BMPH-PTE and DOX-BMPH-MP ND formulations shows that more DOX-BMPH-MP loading quantities do not seem to govern the stability of the MPS1 and ND complex significantly (Figures 4A,B). But the single acyl-chain characteristics of MP may subsequently influence membrane curvature. As the large head groups (DOX) of lipid clusters group together, DOX-BMPH-MPs impose a positive curvature on both sites of the ND membrane. Notably, the MD trajectory of DOX-BMPH-TC ND formulation exhibits the lowest RMSD values and fluctuations (except the case with a drug loading of 25%) than the formerly discussed ND formulations, which can be typically observed in the case with a 50% drug loading ratio (Figure 4C). We speculated that this significant RMSD improvement is primarily attributed to the cholesterol domain of DOX-BMPH-TC, which can stabilize the ND by increasing the order of the lipid packing, enhancing the rigidity, and reducing the fluidity of the membrane. Moreover, DOX-BMPH-TC with fewer rotatable bonds than DOX-BMPH-PTE and DOX-BMPH-MP can also result in reduced structural flexibility and lower RMSD.

### The Electrostatic Interactions between DOX-Prodrug molecules and MSP1 Proteins

**FIGURE 5:**
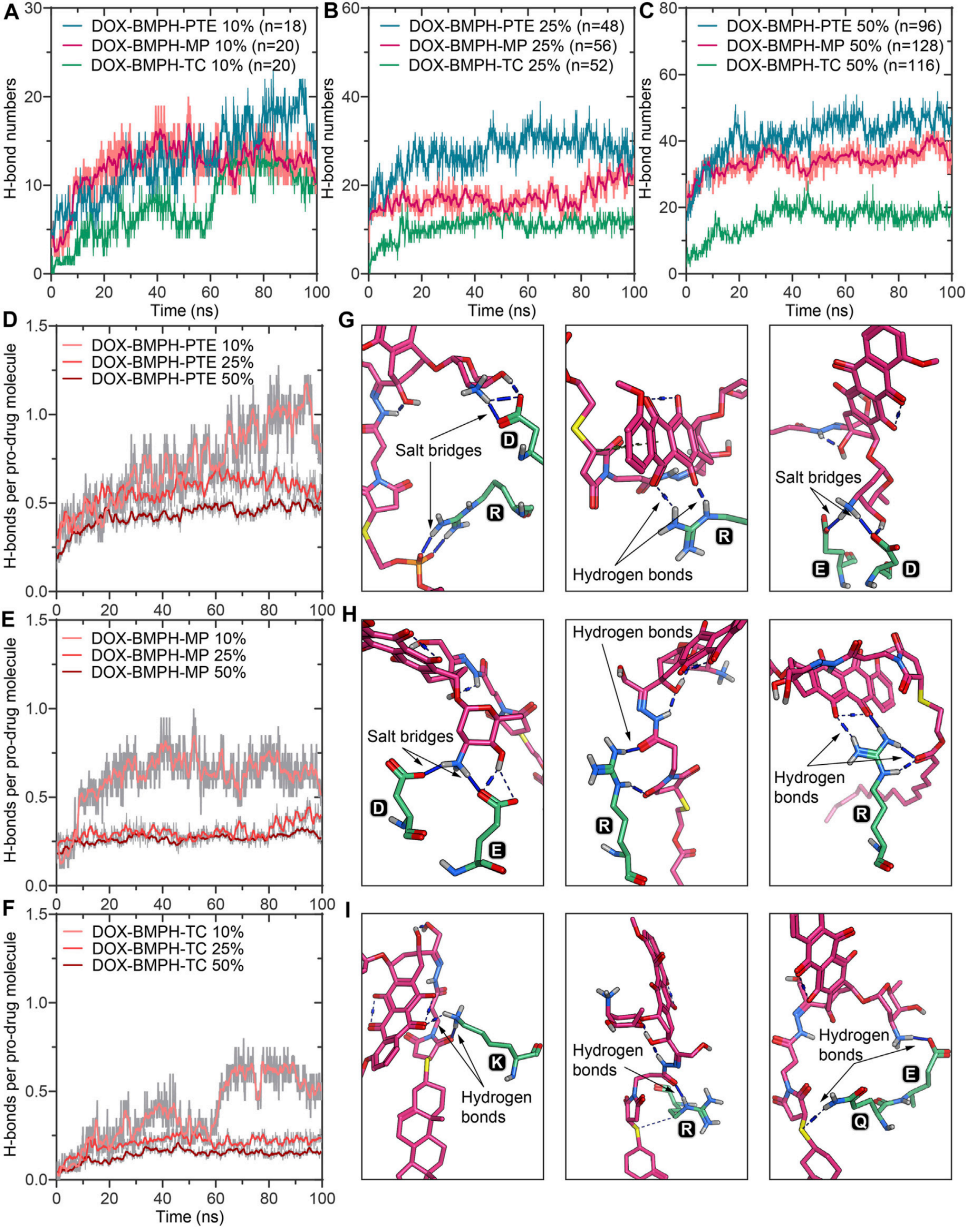
A schematic illustration of the ND formulation designs for free DOX and lipidated DOX-prodrugs.

In MD simulations of DOX-prodrug ND formulations, we observed that the number of hydrogen bonds between DOX-prodrug molecules and MSP1 proteins is proportional to the loading quantities in all types of ND formulations (Figures 5A–C). The DOX-BMPH-PTE ND formulation consistently has the highest average counts of hydrogen bonds (after 50ns of simulation) across different loading quantities (10%, H_n_ = 16.1; 25%, H_n_ = 29.5; 50%, H_n_ = 45.5), followed by a consistent pattern for DOX-BMPH-MP ND formulation (10%, H_n_ = 13.1; 25%, H_n_ = 18.2; 50%, H_n_ = 35.8), and received the lowest average counts for DOX-BMPH-TC ND formulation (10%, H_n_ = 10.3; 25%, H_n_ = 11.5; 50%, H_n_ = 17.8). This regularity difference between the different types of ND formulations could be explained by the fact that the number of rotatable bonds and the structural flexibility of DOX-prodrug molecules are in descending order: DOX-BMPH-PTE > DOX-BMPH-MP > DOX-BMPH-TC (Figure 1). The loss of conformational freedom in DOX-prodrug molecules results in limited DOX-MSP1 interactions, and hence the less number of hydrogen bonds. This implies that increasing in conformational restriction of drug molecules may have a reduced influence on MSP1 dynamics.

The fluctuations of hydrogen bonds per-DOX interaction with MSP1 over the simulation time show that hydrogen bonds per-DOX are significantly reduced with increasing DOX quantities (Figures 5D–F), and the minimum limits are achieved when the proportions of DOX rise to above 25%. In accordance with the center clusters of DOX-BMPH-PTE ND, we found abundant electrostatic interactions between DOX prodrugs and MSP1 proteins, including salt bridges and hydrogen bonds (Figures 5G–I). Similar interactions are observed in DOX-BMPH-MP ND central structures, whereas in DOX-BMPH-TC ND central structures, only a few hydrogen bonds were formed with MSP1 proteins. Collectively, Arg, Lys, Asp, and Glu residues of MSP1 proteins have commonly participated in hydrogen bonding and salt-bridge interactions with DOX-prodrug molecules.

## Solvent Accessible Surface Area and Stability of DOX-Prodrug Loaded ND Assemblies

**FIGURE 6:**
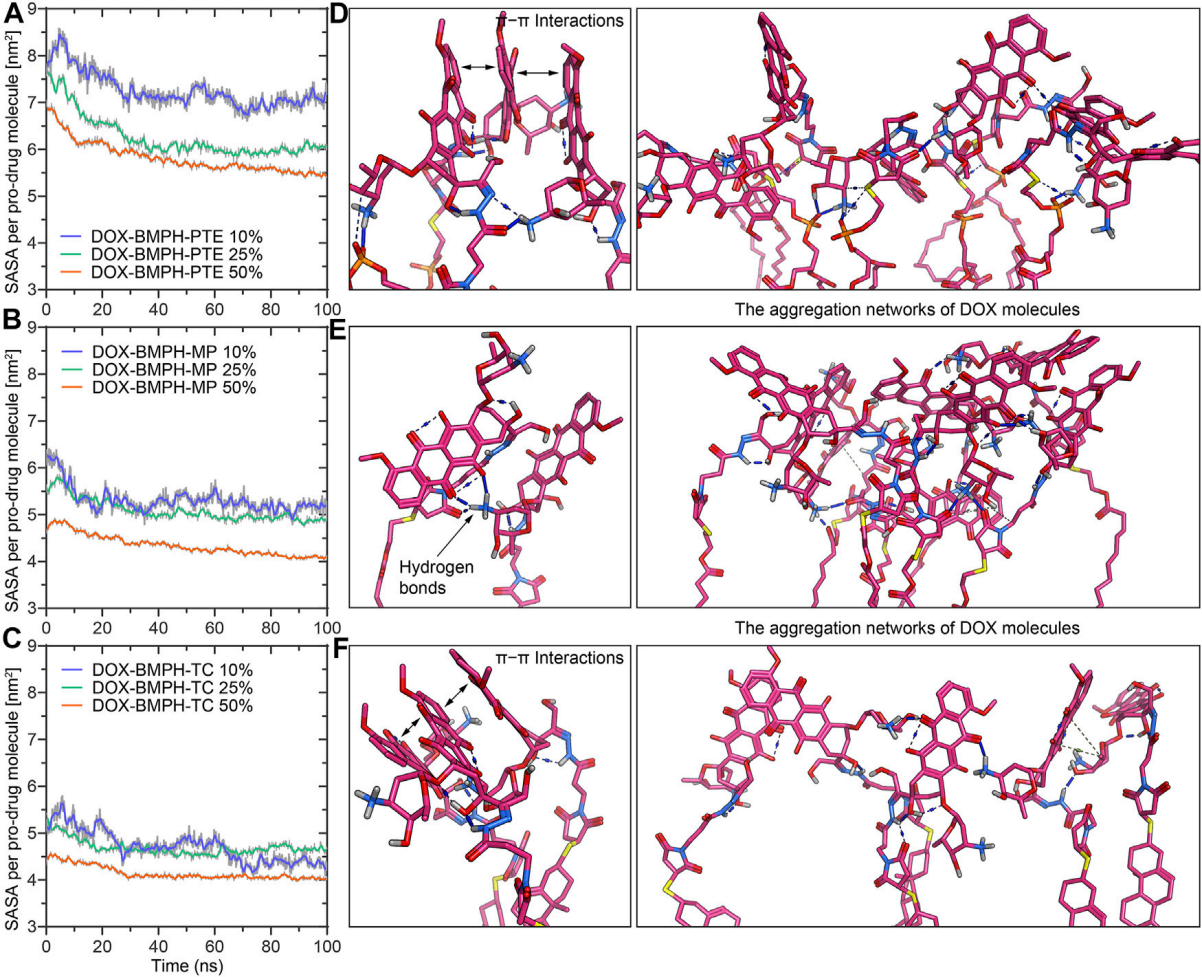
MD simulation analysis of the solvent accessible surface area (SASA) and self-aggregation behavior of DOX-prodrug ND formulations. Time evolution of the SASA per prodrug molecule during 100 ns of MD simulation for ND formulations of **(A)** DOX-BMPH-PTE. **(B) **DOX-BMPH-MP. **(C)** DOX-BMPH-TC. The aggregation networks of DOX molecules in ND formulations of **(D)** DOX-BMPH-PTE. **(E)** DOX-BMPH-MP. **(F)** DOX-BMPH-TC. Overall Stability and Flexibility of MSP1 Protein Assembled with Different DOX-ND Formulations.

Solvent accessible surface area (SASA) of DOX-prodrug loaded ND assemblies could be an important factor in stability studies. The SASA per prodrug molecule of different ND formulations is shown in Figures 6A–C. We noticed that a smaller head moiety of lipidated DOX-prodrugs can lower SASA per prodrug molecule. Besides, comparable trajectories are observed for 10 and 25% of both DOX-BMPH-MP ND and DOX-BMPH-TC ND (Figures 6B,C), which suggests rigidity of the DOX-BMPH-MP and DOX-BMPH-TC head moieties. It is notable that the SASA per prodrug molecule appears to be dropped at high proportions of DOX-prodrug quantity (50%), indicating that the tendency of decreasing in SASA could be governed by the quantity-dependent self-aggregation of DOX (Fülöp et al., 2013). Various interactions such as hydrogen bonding, π-π, π-alkyl stacking, and hydrophobic interaction are frequently involved in the aggregation networks of DOX. The visual inspection of central structures found that the presence of six-membered daunosamine sugar of DOX molecules plays a dominant role in forming hydrogen-bonding networks (Figures 6D–F). Other substructures, including aglycone moiety, phosphate groups, glycerol groups, and maleimide groups, also facilitated the intermolecular and intramolecular electrostatic interactions.

**FIGURE 7:**
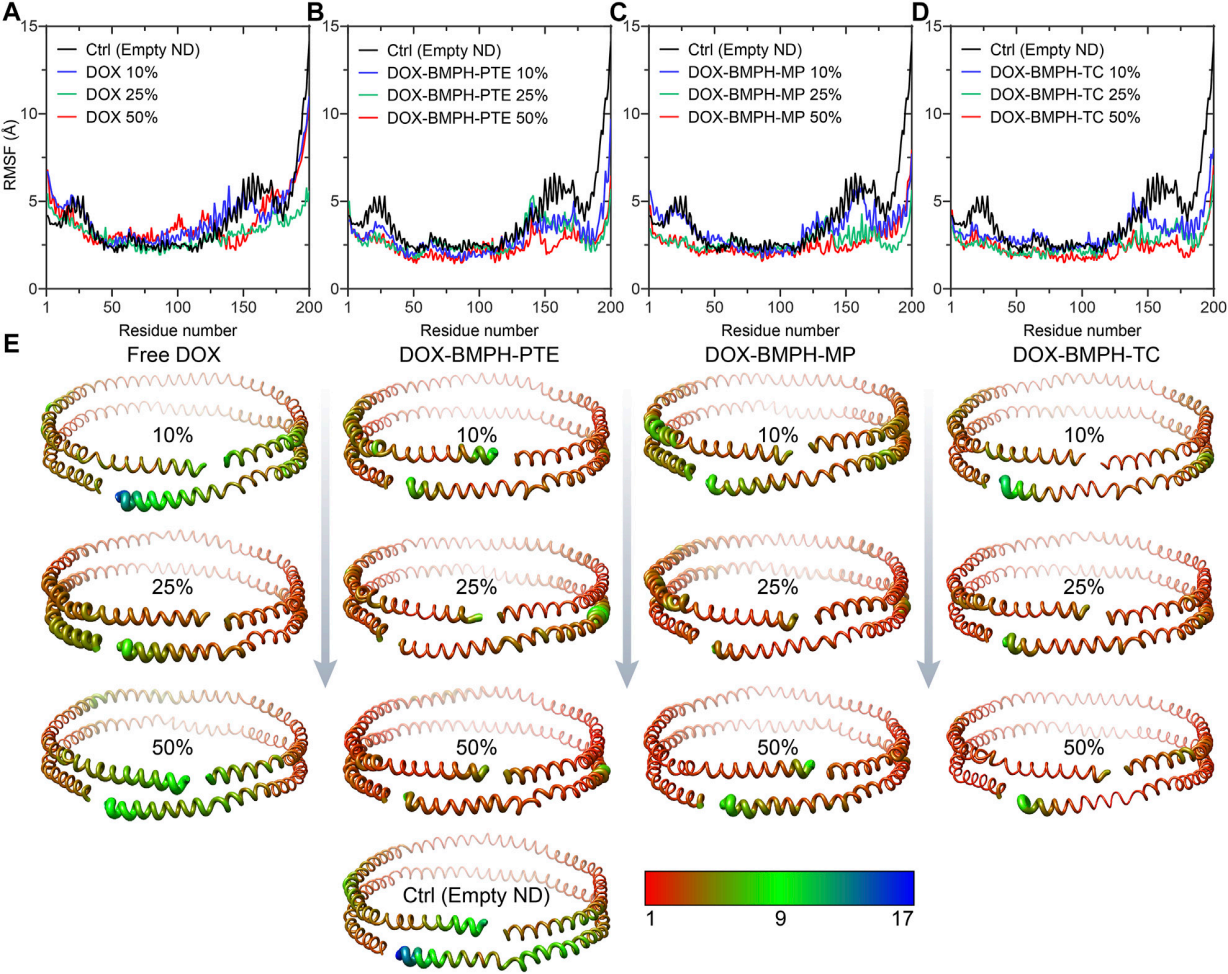
Root Mean Square Fluctuation (RMSF) analyses MSP1 protein assembled with different DOX-ND formulations. Each MSP1 contained 200 residues, and the abscissa in the line charts represents the average value of RMSF in the same residue of two MSP1 proteins. RMSF of four systems compared with MSP1 residues in the empty ND: **(A**) MSP1 residues in the free DOX-ND formulations. **(B)** MSP1 residues in DOX-BMPH-PTE ND formulations. **(C) **MSP1 residues in DOX-BMPH-MP ND formulations. **(D) **MSP1 residues in DOX-BMPH-TC ND formulations. **(E) **Comparison of the fluctuations of each residue between four ND formulations and the empty ND with variants colored and sized according to its RMSF contribution. The coloring-thickness scale varies from deep red and thin (low fluctuations) to dark blue and thick (high fluctuations).

To observe and compare the motion of MSP1 residues, we performed the root mean square fluctuation (RMSF) analyses for both the empty ND (reference structure) and different DOX-ND Formulations (Figure 7). In all cases, the most flexible regions are the C-terminal residues of the MSP1 (Figures 7A–D). As the DOX loading increased, each type of formulation followed a similar tendency with varying degrees of reduction in global RMSF values (Figure 7E). It is observed that residues of the ND formulation of DOX-BMPH-TC are more rigid than other ND formulations. This is in agreement with RMSD results and indicates that there were relatively strong interactions between the lipids/Drugs and MSP1 proteins, as well as lower fluidity of the membrane, which stabilized the DOX-BMPH-TC ND in a more compact conformation than others. Nevertheless, the rigidity of residues could be improved by loading a higher proportion of lipidated-prodrugs except for free DOX ND formulation.

## Conclusion

The molecular dynamic behavior of both free DOX and DOX conjugated lipid prodrug molecules loaded in ND formulations was investigated in depth by a series of all-atom MD simulations. We found that the interactions between free DOX molecules and the ND system are labile due to the strong electrostatic interaction between the amino sugar moiety of DOX and the phosphate head group of DPPC. These hydrogen bonds and intrinsic steric hindrance altered the ideal binding mode of DOX and resulted in an unfavorable orientation of DOX attached on the lipid surface rather than the vertical insertion of the AQ ring. Such amphiphilic characteristics of DOX may enable rapid membrane penetration and diffusion (Toroz and Gould, 2019). The stability of DOX binding modes to the ND membrane could be dependent on lipid composition (Siani et al., 2022). Through the comparison between three types of ND formulations of lipidated-DOX prodrugs, we observed that lipidation approaches could provide sufficient conformational stability for prodrug loading and different lipid used for lipidation led to variable loading capacity. ND formulation of DOX-BMPH-MP may be selected for more drug loading quantities as compared to other formulations. Our results also demonstrated that the global conformational stability of DOX-prodrug-loaded ND Assemblies is influenced by the molecular flexibility and lipid composition of DOX-prodrug molecules. Thus, ND formulation of DOX-BMPH-TC may be considered for better dynamic stability. We have also demonstrated that the increase in proportions of DOX-prodrug within a specific range (e.g., 10–50%) could induce DOX self-aggregation and hydrogen bonding with MSP1 residues, incidentally lowering the membrane fluidity and hence improving the conformational stability of ND formulations.

The main limitation of our study lies in the assumption that ND formulations of DOX satisfy the discoidal double-belt model and can be properly prepared under experimental conditions. Although this study mainly focused on comparing ND formulations with one another, simulated time could be another limitation that affects the accuracy. Besides, the effect of drug loading quantity on ND solubility was not within the scope of the study. Nevertheless, our findings offered a molecular insight into how lipidation of DOX influences the dynamic performance of ND formulations. The obtained information may guide the practical design and optimization of the ND formulation of DOX. The in silico approach adopted here exhibits future potential as a virtual screening or evaluation method to develop ND formulations.

## Data Availability

The raw data supporting the conclusions of this article will be made available by the authors, without undue reservation.
